# Reply: Improving Outcomes of Unstable Patients With Acute Pulmonary Embolism

**DOI:** 10.1016/j.jacadv.2024.101067

**Published:** 2024-07-04

**Authors:** Zach Rozenbaum

I thank Dr Rouleau and colleagues for their response to our paper,^1^ and agree that the current recommendations and guidelines regard systemic thrombolysis (ST) as first-line therapy for unstable pulmonary embolism (PE) patients. Yet, the risk-benefit ratio is far from robust due to high bleeding rates.^1^ Moreover, studies investigating high-risk PE did not consistently report efficacious results;^2^ hence, the viewpoint aimed to challenge guidelines.^1^ Furthermore, guidelines are not in line with real-world practice, as 88.7% of unstable PE patients do not receive ST,^1^ thus reflecting the risky nature of ST therapy. Since ST has been available for many years, the extent of its use is unlikely to increase, and newer therapies should be pursued in order to attempt to improve the presently suboptimal outcomes of patients with PE.^3^

An important point made by Dr Rouleau and colleagues is that not all centers have access to catheter-directed therapies. This could be a result of the guidelines that mention catheter-directed therapies as one of the alternative options. A strong recommendation may encourage more widespread availability. Dr Rouleau and colleagues also mention the importance of further studies on novel dosing regimens. Notably, most ongoing or planned randomized studies do not use ST as the control group.^4^

When the standard of treatment has high complication rates, a replacement should be considered, leaving ST as the alternative and not vice versa. The discrepancy between the treatment workflow of centers with access to catheter-directed therapies and the guidelines highlights the need for widespread awareness of newer effective options for treating PE.
